# Puberty Timing and Sex-Specific Trajectories of Systolic Blood Pressure: a Prospective Cohort Study

**DOI:** 10.1161/HYPERTENSIONAHA.121.18531

**Published:** 2022-05-19

**Authors:** Kate N. O’Neill, Joshua A. Bell, George Davey Smith, Kate Tilling, Patricia M. Kearney, Linda M. O’Keeffe

**Affiliations:** School of Public Health, University College Cork, Ireland (K.N.O.N., P.M.K., L.M.O.K.).; MRC Integrative Epidemiology Unit at the University of Bristol, United Kingdom (J.A.B., G.D.S., K.T., L.M.O.K.).; Population Health Sciences, Bristol Medical School, University of Bristol, Bristol, United Kingdom (J.A.B., G.D.S., K.T., L.M.O.K.).

**Keywords:** blood pressure, cardiovascular diseases, puberty, risk factor

## Abstract

**Background:**

Sex differences in systolic blood pressure (SBP) emerge during adolescence but the role of puberty is not well understood. We examined sex-specific changes in SBP preceding and following puberty and examined the impact of puberty timing on SBP trajectories in females and males.

**Methods:**

Trajectories of SBP before and after puberty and by timing of puberty in females and males in a contemporary birth cohort study were analyzed. Repeated measures of height from age 5 to 20 years were used to identify puberty timing (age at peak height velocity). SBP was measured on ten occasions from 3 to 24 years (N participants, 4062; repeated SBP measures, 29 172). Analyses were performed using linear spline multilevel models based on time before and after puberty and were adjusted for parental factors and early childhood factors.

**Results:**

Mean age at peak height velocity was 11.7 years (SD, 0.8) for females and 13.6 years (SD, 0.9) for males. Males had faster rates of increase in SBP before puberty leading to 10.19 mm Hg (95% CI, 6.80–13.57) higher mean SBP at puberty which remained similar at 24 years (mean difference, 11.43 mm Hg [95% CI, 7.22–15.63]). Puberty timing was associated with small transient differences in SBP trajectories postpuberty in both sexes and small differences at 24 years in females only.

**Conclusions:**

A large proportion of the higher SBP observed in males compared with females in early adulthood is accrued before puberty. Interventions targeting puberty timing are unlikely to influence SBP in early adulthood.

**Novelty and Relevance**

**What Is New?**

This research uses an objective measure of puberty timing and repeated systolic blood pressure (SBP) measurements from ages 3 to 24 years to explore changes in SBP among females and males before and after puberty and to examine the association between puberty timing and SBP trajectories from infancy to early adulthood.

**What Is Relevant?**

The time before puberty is an important period for the emergence of higher SBP in males compared with females. Puberty timing itself is not strongly associated with SBP in early adulthood.

**Clinical/Pathophysiological implications**

Interventions targeting puberty timing are unlikely to greatly influence SBP in females and males in early adulthood.

High systolic blood pressure (SBP) is a leading modifiable risk factor for cardiovascular disease.^1–3^ SBP tracks from childhood through to adulthood^4^; both higher levels of SBP and faster rates of increase in SBP during adolescence are positively associated with the risk of developing hypertension in later life.^5^ Sex differences in SBP across the life course are well established with males having higher SBP than females throughout much of adult life until mid to later life when steeper rises in SBP are observed in females.^6–8^ Sex differences in SBP emerge during adolescence and by age 18 there is evidence of 10 mm Hg higher SBP in males compared with females.^6,9–11^

Puberty has been identified as a crucial period in adolescence which may account for the emergence of a sex difference in SBP with the disparate action of sex steroids on blood pressure put forward as a biological mechanism.^10,12,13^ However, few studies to date have examined and compared change in SBP before and after puberty in females and males.^13,14^ In an analysis with repeated measures of SBP over a 10-year period from before to after puberty, males had higher SBP than females and similar patterns of change throughout the time observed, albeit with faster rates of increase in SBP in males around the pubertal growth period.^14^ However, the study included only 182 participants, not all of whom were followed up into early adulthood, limiting insights into the role of puberty in SBP change after more transient effects on SBP at puberty subside.

In addition to the potential role of puberty in the emergence of sex differences in SBP, several studies have examined whether puberty timing influences SBP later in adulthood.^15–19^ However, results have been largely inconsistent with some studies demonstrating associations between early puberty and higher SBP in both sexes^15,16^ while others document associations in males but not females^17,18^ or provide no strong evidence of associations in either sex.^19^ These studies have been limited by their use of self-report measures of puberty timing or have lacked data on prepubertal adiposity gain, an important confounder of puberty timing—cardiovascular risk associations.^19,20^ In addition, these studies only examined single measurements of SBP in adulthood. Understanding whether puberty timing is associated with SBP trajectories before and after puberty may provide further insights into the potential causality of associations between puberty timing and SBP in adulthood among females and males. Any observed associations of puberty timing with SBP early in childhood before puberty are unlikely to reflect a causal effect as this is temporally implausible and, therefore, likely explained by confounding or possibly shared genetic architecture between SBP and puberty timing. Consequently, if puberty timing is associated with SBP measured early in childhood (before puberty) to a similar degree as SBP measured in adulthood (after puberty), this would suggest that puberty timing itself is unlikely to be a cause of SBP.

Using an objective growth-based measure of puberty (age at peak height velocity [aPHV]), repeated SBP measures from 3 to 24 years of age from a large contemporary prospective birth cohort study in the southwest of England and with adjustment for prepubertal adiposity gain, we first examine change in SBP before and after puberty to better understand whether sex-specific changes in SBP precede or follow puberty. Second, we examine the association between puberty timing and SBP trajectories before and after puberty in females and males, to gain a better understanding of the likely causality of associations between puberty timing and SBP in adulthood.

## Methods

### Study Participants

Data were from first-generation offspring of ALSPAC (Avon Longitudinal Study of Parents and Children), a population-based prospective birth cohort study in southwest England.^21,22^ Because of the sensitive nature of the data collected for this study, requests to access the data from qualified researchers trained in human subject confidentiality protocols may be sent to the University of Bristol at www.bristol.ac.uk/alspac/researchers/access. Pregnant women resident in one of the 3 Bristol-based health districts with an expected delivery date between April 1, 1991 and December 31, 1992 were invited to participate. The study is described elsewhere in detail.^21–23^ ALSPAC initially enrolled a cohort of 14 451 pregnancies, from which 14 062 live births occurred and 13 988 children were alive at 1 year of age. When the oldest children were aged ≈7 years, an attempt was made to bolster the initial sample with eligible cases who had not joined the study originally. Therefore, the total sample size for analyses using any data collected after the age of 7 years is 15 454 pregnancies, resulting in 15 589 foetuses. Of these 14 901 were alive at 1 year of age. Follow-up has included parent- and child-completed questionnaires, research clinic attendance, and links to routine data. Data gathered from participants at 22 years of age and onwards were collected and managed using Research Electronic Data Capture electronic data capture tools.^24,25^ Ethical approval for the study was obtained from the ALSPAC Ethics and Law Committee and the Local Research Ethics Committees. Informed consent for the use of data collected via questionnaires and clinics was obtained from participants following the recommendations of the ALSPAC Ethics and Law Committee at the time. The study website contains details of all the data that is available through a fully searchable data dictionary http://www.bristol.ac.uk/alspac/researchers/our-data/.

### Data

#### Assessment of Puberty Timing

Puberty is a period of intense hormonal activity and rapid growth, of which the most striking feature is the spurt in height.^26^ aPHV is a validated measure of pubertal timing^26^ captured using Superimposition by Translation and Rotation, a nonlinear multilevel model with natural cubic splines which estimates the population average growth curve and departures from it as random effects.^27,28^ Using Superimposition by Translation and Rotation, PHV was identified in ALSPAC participants using numerical differentiation of the individually predicted growth curves, with aPHV being the age at which the maximum velocity is observed.^27–29^ Repeated data on measured height from research clinics were used here to derive aPHV. Individuals with at least one measurement of height from 5 to <10 years, 10 to <15 years, and 15 to 20 years were included. Data were analyzed for females and males separately. The model was fitted using the Superimposition by Translation and Rotation package in R version 3.4.1. Further details of height measures are included in Table S1, and information on how aPHV was derived is described elsewhere^29^ and in Methods S1.

#### Measurement of SBP

Ten measurements of SBP (mean ages 3, 5, 7, 9, 10, 11, 12, 15, 18, and 24 years) were available from research clinic assessments. In a random 10% of the cohort, SBP was measured at Children in Focus clinical assessments conducted in early childhood (ages 3, 4, 5 years).^21^ After this (from 7 to 24 years), all children were invited to attend focus clinics. At each clinic, SBP was measured at least twice each with the child sitting and at rest with the arm supported, using a cuff size appropriate for the child’s upper arm circumference and a validated blood pressure monitor. The mean of the 2 final measures was used. Further details are provided in Methods S2.

#### Measurement of Covariates

We selected potential confounders a priori and used a directed acyclic graph to illustrate our causal assumptions related to this research question.^30^ We considered the following as potential confounders of the association between puberty/age at puberty and SBP: birth weight, gestational age, maternal education, mother’s partner’s education, parity, maternal smoking during pregnancy, maternal age, maternal prepregnancy body mass index (BMI), household social class, marital status, ever breastfed (all measured by mother-completed questionnaires) and prepubertal gains in BMI from one up to 9 years of age. Further details of measurements are available in Methods S3.

### Sample Size for Analysis

Participants who had an estimate of aPHV, at least one measure of SBP from 3 to 24 years and complete data on all confounders were included in analyses, leading to a total sample of 4062 (2139 females and 1923 males) with 10 839 participants excluded (Figure S2). Participants who reported being pregnant at the 18-year clinic or 24-year clinic were excluded from the multilevel models at that timepoint (N=9).

### Statistical Analysis

#### Pubertal Age-Based Multilevel Model

Linear spline multilevel models were used to examine change in SBP during childhood and adolescence and allow for the inclusion of participants with at least one SBP measurement throughout the follow-up period.^31,32^ A common approach to modeling change over time using multilevel models involves examining change by chronological age.^9,33^ However, when change before or after a specified event is of interest (eg, puberty or menopause), it is also possible to model change according to other time metrics such as time before and after the event.^20^ Thus, to gain a greater understanding of the role of puberty and its timing in change in SBP during childhood and adolescence, we modeled trajectories of SBP by time before and after puberty. The final model for females had 4 periods of SBP change: one prepubertal period and 3 postpubertal periods. In males, the final model for change in SBP also had 4 periods of change: 2 prepubertal periods and 2 postpubertal periods. Due to different periods of change in females and males all models were sex stratified. Further details on the selection of models and model fit are included in Methods S3, Table S2 and Table S3.

To explore sex-specific change in SBP before and after puberty, we compared SBP trajectories for the median female (aPHV=11.6 years) and male (aPHV=13.6 years); this provided insight into the sex-specific changes in SBP preceding and following puberty in females and males. As a female with the median aPHV is younger chronologically than the median male and SBP increases with age, we also compared SBP trajectories for a female and male with similar aPHV (age 12.8 years in females [90th percentile] and 12.4 years in males [10th percentile]). This provided insights into whether any differences in trajectories, particularly differences in SBP at puberty between the median female and male were independent of differences in chronological age. We compared the difference in SBP between females and males at age 3 years, at puberty and age 24 years by calculating the mean difference between the sexes and using the pooled standard error to calculate 95% CIs for the difference.

We then examined the effect of aPHV on SBP trajectories before and after puberty in females and males separately. Differences in the rate of change in SBP before and after puberty by aPHV were explored by including an interaction between centred sex-specific aPHV and the intercept (SBP at puberty) and each linear spline period. Figures presented compare SBP trajectories for the median, 10th and 90th sex-specific percentiles of aPHV. Differences in trajectories for a one-year later aPHV are reported in tables. The effect of aPHV on SBP trajectories at age 3 years served as a negative control analysis. Any observed associations of aPHV with SBP early in childhood before puberty cannot be caused by aPHV and are likely explained by confounding, particularly by adiposity, or possibly shared genetic architecture between SBP and puberty timing.

Confounders were included as interactions with the intercept and each linear spline period. To account for the confounding effect of prepubertal adiposity gain, individual-specific residuals derived from multilevel models of BMI from 1 up to 9 years of age were included as interactions with the intercept and each linear spline period. These residuals capture each individual’s deviation from the average BMI trajectory.^34^ Details on multilevel models of BMI are provided in Methods S3 and have been published previously.^34^ Analyses were performed with and without adjustment for confounders.

#### Additional and Sensitivity Analyses

We performed weighted sensitivity analyses using inverse probability weighting to address potential selection bias due to exclusion based on missing data. The individual level weights were estimated using a logistic regression model that included all listed socio-demographic characteristics (sex, birth weight, gestational age, maternal education, mother’s partner’s education, parity, maternal smoking during pregnancy, maternal age, maternal prepregnancy BMI, household social class, marital status, ever breastfed, and prepubertal gains in BMI) and were incorporated into the multi-level models.^35^ We additionally performed unadjusted analyses on the sample of participants that had data on aPHV and at least one measure of SBP from 3 to 24 years; this analysis included an additional 1640 participants excluded from our main analysis due to missing confounder data and provided insight into potential selection bias due to missing confounder data. We also explored the robustness of our findings to the number and timing of SBP measures (by restricting the sample to participants with at least one SBP measure before and one after aPHV and to those with a minimum of 5 SBP measures in total during follow-up) and to the pubertal age modeling strategy (by comparing results from models using chronological age-based trajectories of SBP). Finally, we conducted additional analyses adjusting for fat mass at age 9 years to further explore the confounding role of adiposity and a further analysis adjusting for lean mass at age 9 years to explore the role of body mass. Further details on these analyses are provided in Methods S4.

## Results

The characteristics of participants included in analyses, by sex, are shown in Table 1. Similar socio-demographic characteristics were observed for females and males. Mean aPHV was 11.7y (SD=0.8) among females and 13.6 years (SD=0.9) among males. Mothers of participants included in the analysis were more likely to be married, have higher household social class, higher education, higher partner education, lower prevalence of smoking during pregnancy, lower parity and higher maternal age compared with mothers of participants excluded due to missing exposure, outcome, or confounder data (Table S4). However, aPHV and SBP were similar between included and excluded participants (Table S4).

**Table 1.**
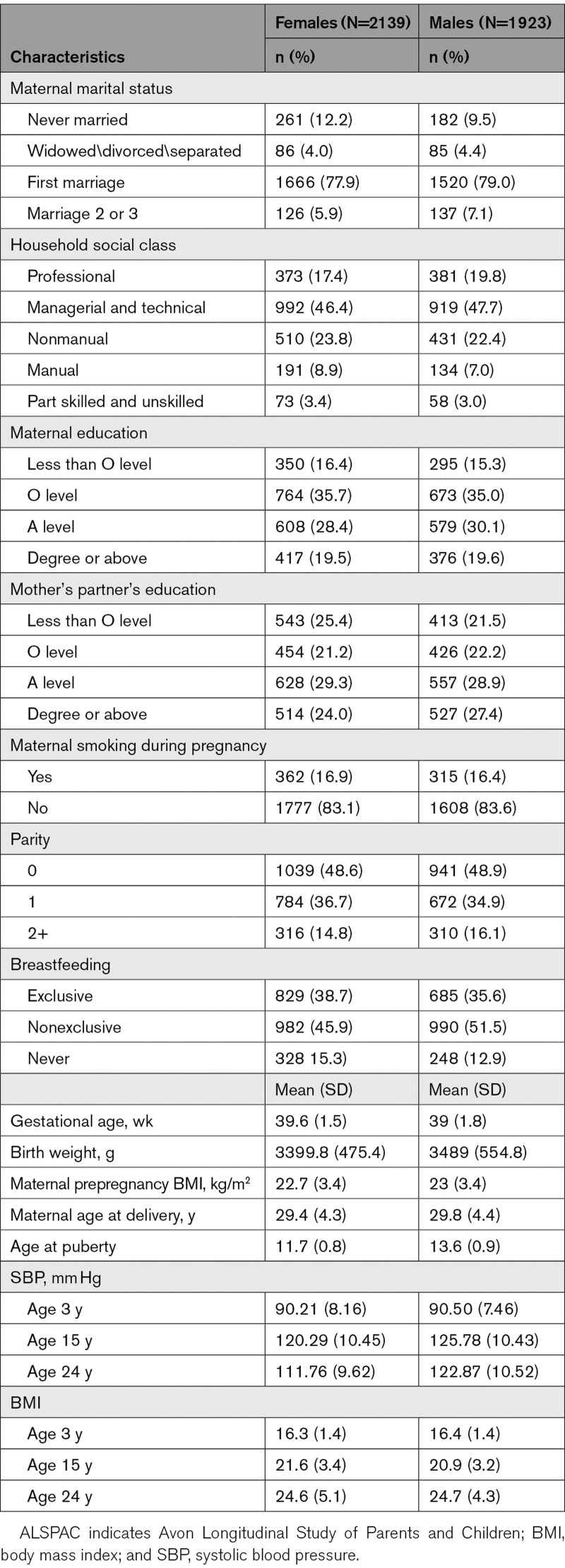
Characteristics of ALSPAC Participants Included in the Analysis, by Sex

### Change in SBP Before and After Puberty

**Figure 1.**
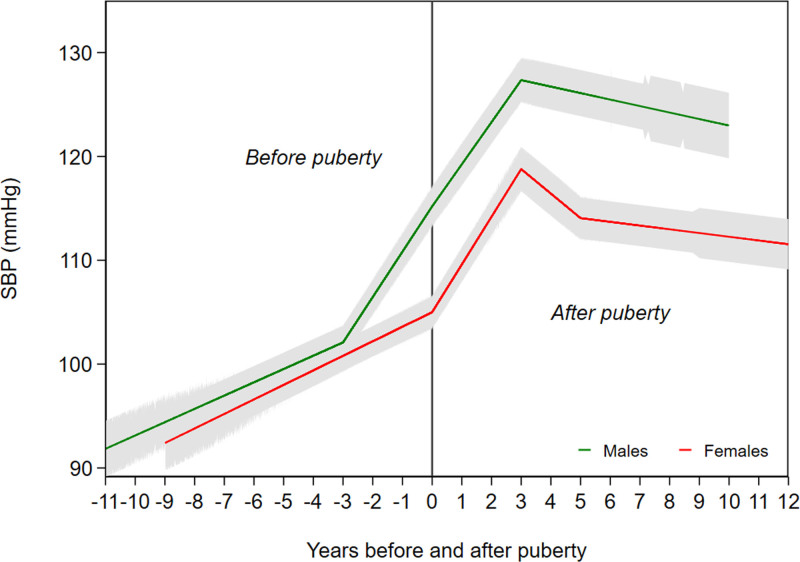
**Mean adjusted trajectories of systolic blood pressure (SBP) in females and males before and after puberty from multilevel models based on pubertal age.** Models are adjusted for birth weight, gestational age, maternal education, parity, maternal smoking during pregnancy, maternal age, maternal prepregnancy body mass index (BMI), household social class, marital status, partner education, breastfeeding, BMI residuals of offspring.

Mean adjusted trajectories of SBP before and after puberty in females and males at the median aPHV are presented in Figure 1. In adjusted models, females and males had similar SBP at age 3 years (Figure 1, Table 2). At puberty (median age 13.6 years in males and 11.7 years in females), males had a 10.19 mm Hg (95% CI, 6.80–13.57) higher SBP compared with females (Table S5). By 24 years, this difference increased to 11.43 mm Hg (95% CI, 7.22–15.63). Higher SBP at puberty in males appeared to be attributable to steep increases in SBP in males in the 3 years before puberty (Table 2). Mean adjusted SBP trajectories for females and males of similar aPHV (age 12.8 years in females [90th percentile] and 12.4 years in males [10th percentile]) are shown in Figure S2; at puberty males had a 5.75 mm Hg (95% CI, 2.30–9.20) higher SBP compared with females; this difference increased to 10.83 mm Hg (95% CI, 6.41–15.25) higher SBP in males compared with females at 24 years of age (Table S5).

**Table 2.**
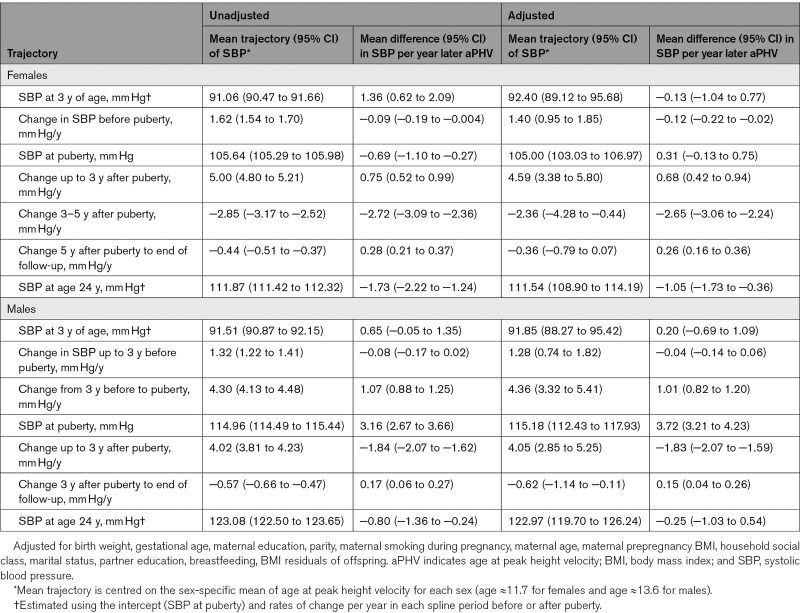
Unadjusted and Adjusted Mean Trajectory and Mean Difference in Trajectory of SBP Per Year Later Age at Peak Height Velocity, From Pubertal Age Multilevel Models

### Puberty Timing and SBP

#### Females

**Figure 2.**
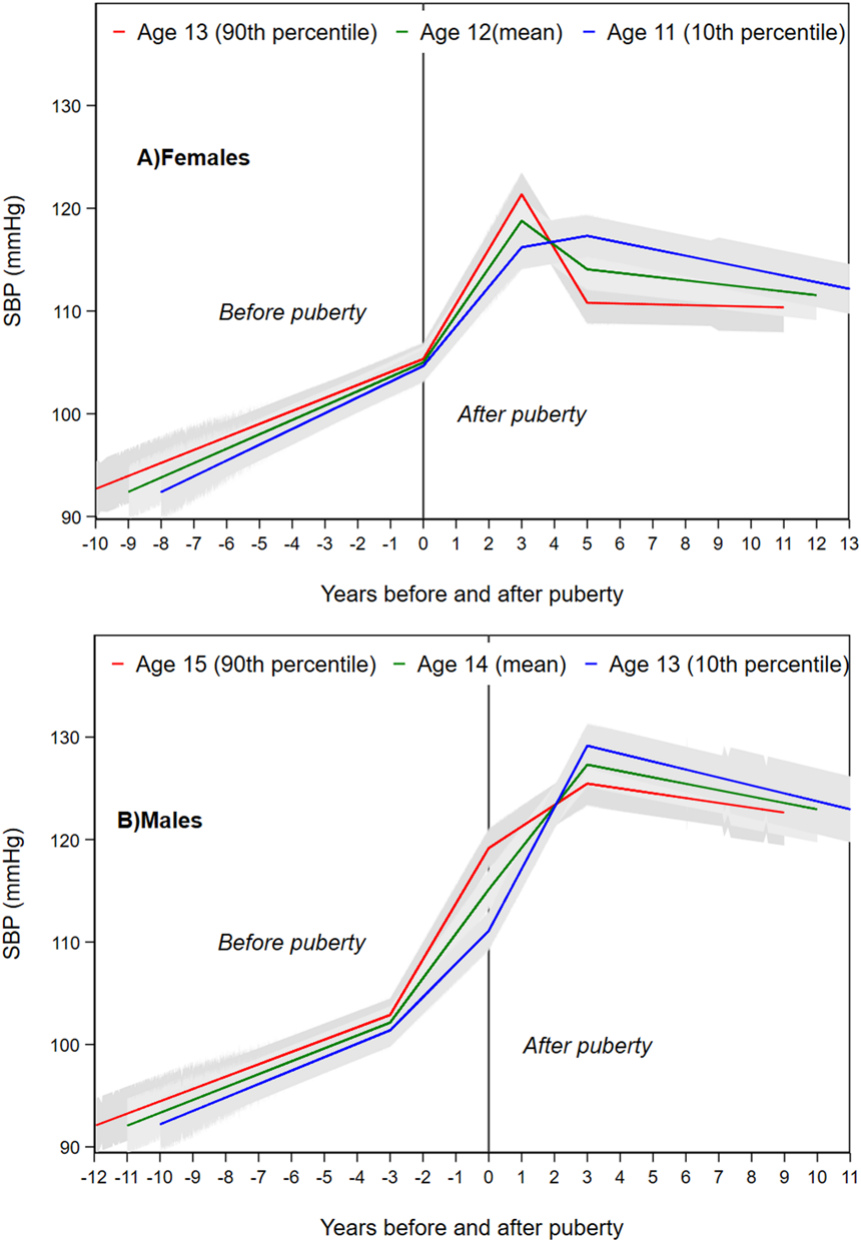
**Mean adjusted trajectories of systolic blood pressure (SBP) in females and males for the 10th, median and 90th sex-specific percentiles of age at peak height velocity from multilevel models based on pubertal age.** Ages presented are rounded exact ages are 12.8 y, 11.7 y, and 10.7 y for females and 14.7 y, 13.6 y, and 12.4 y for males. Models are adjusted for birth weight, gestational age, maternal education, parity, maternal smoking during pregnancy, maternal age, maternal prepregnancy body mass index (BMI), household social class, marital status, partner education, breastfeeding, BMI residuals of offspring.

Mean adjusted female trajectories of SBP for the 10th (age 11 years), 50th (age 12 years), and 90th (age 13 years) percentiles of aPHV are presented in Figure 2. In adjusted models, there was no evidence of an association between a one-year later aPHV and SBP at 3 years of age (difference, −0.13 mm Hg [95% CI, −1.04 to 0.77]) or SBP at puberty (difference, 0.31 mm Hg [95% CI, −0.13 to 0.75]; Table 2). A one-year later aPHV was associated with faster increases in SBP in the 3 years’ postpuberty and faster decreases in SBP from 3 to 5 years after puberty. From 5 years after puberty to the end of follow-up, a one-year later aPHV was associated with a 0.26 mm Hg (95% CI, 0.16–0.36) per year slower decrease in SBP. By age 24 years, a one-year later aPHV was associated with a 1.05 mm Hg lower SBP (95% CI, −1.73 to −0.36).

#### Males

Mean adjusted male trajectories of SBP for the 10th (age 13 years), 50th (age 14 years), and 90th (age 15 years) percentiles of aPHV are presented in Figure 2. Similar to females, in adjusted models there was no evidence of an association between aPHV and SBP at 3 years of age (difference, 0.20 mm Hg [95% CI, −0.69 to 1.09]) or in rates of change in SBP from 3 years of age to 3 years’ prepuberty (difference, −0.04 mm Hg per year [95% CI, −0.14 to 0.06]). In the 3 years before puberty, a one-year later aPHV was associated with a 1.01 mm Hg (95% CI, 0.82–1.20) faster increase in SBP per year. At puberty, a one-year later aPHV was associated with 3.72 mm Hg (95% CI, 3.21–4.23) higher SBP. In the 3 years after puberty, a one-year later aPHV was associated with 1.83 mm Hg (95% CI: −2.07 to −1.59) slower increases per year and 0.15 mm Hg (95% CI, 0.04–0.26) slower decreases per year in the period from 3 years’ postpuberty to the end of follow-up. By 24 years of age, there was no evidence of a difference in SPB per year later aPHV (difference, −0.25 mm Hg [95% CI, −1.03 to 0.54]).

### Additional and Sensitivity Analyses

When analyses were conducted in the full sample of participants rather than those with complete confounder data, results were comparable (Table S6) as were results from the inverse probability weighted analyses (Table S7). Results were not appreciably different when analyses were restricted to participants with at least one measure of SBP before and one measure after aPHV, or to participants with at least five measurements of SBP (Table S8 and S9). Results were also similar in chronological age-based models (Table S10). Adjusting for DXA fat mass at age 9 years also resulted in similar results (Tables S11 and S12) as did adjustment for lean mass at age 9 years (Tables S13 and S14).

## Discussion

In this prospective cohort study, the largest to date with an objective height-based measure of puberty timing and repeat assessments of SBP from 3 to 24 years of age, we aimed to better understand the role of puberty and its timing in sex-specific trajectories of SBP across the early life course. Our findings suggest that a large proportion of the sex difference in SBP in early adulthood is accrued before puberty with the remainder arising in the 5-year period postpuberty. These findings suggest that prevention of sex differences in SBP in adulthood may benefit from a life course approach starting before puberty. Our results on puberty timing and SBP trajectories before and after puberty demonstrated no strong evidence of associations suggesting that puberty timing itself is unlikely to impact SBP in adulthood.

### Comparison With Other Studies

Previous life course analyses of SBP trajectories document a maximum sex difference at age 26 years with higher SBP in males compared with females.^6^ Our findings suggest that a large proportion of this sex difference is established before puberty with the remainder accruing in the 5-year period postpuberty, regardless of whether we compare females and males of the sex-specific median age at puberty or the same ages at puberty. These results are broadly consistent with other prospective studies.^13,14^ A US study (n=182) examining rates of SBP change before and after puberty, defined using peak growth velocity, showed that SBP was higher in males compared with females at any given age from 5 to 25 years.^14^ Similar to our findings, males had nearly 8 mm Hg higher SBP at puberty compared with females and rates of change in SBP were more pronounced in males with larger increases observed around the pubertal growth period. This is also consistent with previous studies documenting increasing SBP in males during adolescence compared with females.^10,12^

Our findings suggested small and relatively transient associations of aPHV with SBP trajectories post puberty. By age 24 years, and after adjustment for early childhood BMI, aPHV was associated with only small differences in SBP in females and no differences in males. A Mendelian randomisation study also conducted in ALSPAC (n=3611) found no strong evidence of associations between puberty timing (measured using reported age at menarche or voice breaking) and SBP at 18 years of age in either females or males, after adjusting for BMI measured at age 8 years.^19^ Results were similar to our findings with overlapping CIs between the estimates in both studies. Our findings build on this evidence using an objective measure of puberty timing to reduce measurement error and improve consistency of measurement between females and males. Furthermore, using measures of height to estimate puberty timing increased both the sample size and minimized the potential for selection bias in our study compared with relying on self-report puberty questionnaires with only modest response rates. Our findings are consistent with several other previous studies which also demonstrated slightly lower SBP in females with later puberty timing.^15,36,37^ For instance, a longitudinal analysis of 391 females between the ages of 8 and 21 years in Finland showed a 1.24 mm Hg lower SBP per year later age at menarche.^36^ Our findings are also comparable with a recent sibling analysis in the Scottish Family Health Study (n=7770) that found that later menarche was associated with a lower SBP in adulthood of a similar magnitude.^37^ In addition, a recent systematic review and meta-analysis of eight studies found lower SBP among women with later menarche, though confidence intervals spanned the null value.^16^ However, the association did strengthen when limited to high quality studies suggesting that methodological issues including heterogeneity in the definition of early menarche and small sample sizes influenced the observed association. In contrast to our findings, two studies from a British birth cohort showed some evidence of lower SBP in males late to puberty but no association in females at ages 53 and 60 to 64 years.^17,18^ Measurement error may have influenced the results observed in females with puberty timing measured using mothers’ reports of age at menarche or self-report age at menarche collected when women were 48 years old while, in males, physical examinations at 15 years of age were used to categorize participants into groups of maturity stages.

Previous analysis in ALSPAC demonstrated associations of prepubertal fat mass with puberty timing in both females and males suggesting that the prevention of adiposity in childhood is key for prevention of early puberty, adult adiposity and associated cardiovascular risk.^20^ After adjustment for prepubertal adiposity, associations between puberty timing and SBP attenuated and we conclude that puberty timing itself is unlikely to impact SBP in adulthood. This agrees with other work in ALSPAC which has shown that adiposity in childhood and adolescence is associated with SBP at age 18 years^38^ and age 25 years.^39^ However further analyses are required to extend this work to examine whether prepubertal adiposity is the important driver of these associations and to explore the mechanisms through which adiposity may affect SBP. In addition, the underlying mechanisms for the emergence of a sex difference in SBP at puberty require further exploration. One plausible mechanism is the differential changes in body mass composition in females and males as they transition into puberty.^14^ While our findings show that prepubertal body composition does not account for the observed sex differences in SBP, the role of changing fat mass and lean mass compositions during adolescence and early adulthood in females and males and its impact on SBP throughout this period warrants further exploration. A recent study conducted in ALSPAC found that increases in lean mass and fat mass throughout adolescence and young adulthood were associated with higher SBP at 25 years and associations were stronger in males than females.^39^ Determining the driving factors of the emerging sex difference in SBP is essential in identifying whether interventions are appropriate or even necessary.

### Strengths and Limitations

The main strengths of our study include its prospective design, relatively large sample size, availability of repeated SBP measures from childhood to early adulthood and use of an objective measure of puberty timing (aPHV) in both sexes. A clinical assessment of Tanner staging is the gold standard measure for puberty but was not measured in ALSPAC.^40^ However, aPHV, captured using the Superimposition by Translation and Rotation method, is an objective, validated and noninvasive measure of pubertal timing that is considered the most appropriate and accurate measurement in longitudinal observational studies.^27,41^ The use of this measure to assess puberty timing minimizes measurement error and reduces selection bias when compared with other self-assessment measures of puberty timing.^41,42^ Childhood adiposity is an important confounder of the association between puberty timing and SBP. To account for this, we used individual-level residual estimates from multilevel models of repeated measures of BMI from one up to 9 years of age for adjustment, reducing likelihood of residual confounding by early childhood weight gain in our analysis. While it is plausible that BMI residual estimates are not independent of height and may have resulted in overadjustment in our models, additional analyses adjusting for fat mass at age 9 years as an alternative measure of adiposity (independent of height) found similar results indicating that this was not likely a concern in our analyses. There are also a number of limitations. Participants excluded from the analysis due to missing data or attrition from the cohort were more socially disadvantaged than those included in our analysis leading to potential selection bias and generalisability issues. However, we aimed to minimize potential selection bias by including all participants with at least one measurement of height from 5 to <10 years, 10 to <15 years, and 15 to 20 years to estimate aPHV and at least one measure of SBP from age 3 to 24 years for estimation of SBP trajectories. In addition, though some socio-demographic characteristics differed between included and excluded participants, aPHV and SBP were similarly distributed, thus minimizing the impact of selection bias driven by missing exposure and outcome data in our analysis. Results from weighted sensitivity analyses and analyses with and without selection on complete confounder data were highly similar to the main findings, further indicating a low likelihood of selection bias driven by missing confounder data. Finally, the majority of our cohort were of White European ethnicity. Therefore, our findings may not be generalizable to non-White ethnicities.

### Perspectives

A large proportion of the higher SBP observed in males compared with females in early adulthood is accrued before puberty. The causes of the emerging sex difference in SBP during this period should be examined in future work. Puberty timing was associated with small transient differences in SBP trajectories postpuberty in both sexes with no strong evidence of associations between puberty timing and SBP by early adulthood. Our results indicate that interventions targeting puberty timing are unlikely to greatly influence SBP in females and males in early adulthood.

## Article Information

### Acknowledgments

We are extremely grateful to all the families who took part in this study, the midwives for their help in recruiting them, and the whole ALSPAC (Avon Longitudinal Study of Parents and Children) team, which includes interviewers, computer and laboratory technicians, clerical workers, research scientists, volunteers, managers, receptionists, and nurses. Individual-level ALSPAC data are available following an application. This process of managed access is detailed at www.bristol.ac.uk/alspac/researchers/access. Cohort details and data descriptions for ALSPAC are publicly available at the same web address.

### Sources of Funding

The UK Medical Research Council and Wellcome (grant ref: 217065/Z/19/Z) and the University of Bristol provide core support for ALSPAC (Avon Longitudinal Study of Parents and Children). A comprehensive list of grants funding is available on the ALSPAC website (http://www.bristol.ac.uk/alspac/external/documents/grant-acknowledgements.pdf); this research was specifically funded Wellcome Trust and MRC (grant ref: 076467/Z/05/Z and 086676/Z/08/Z). Dr O’Neill is supported by a Health Research Board (HRB) of Ireland Emerging Investigator Award (EIA-FA-2019-007 SCaRLeT). Dr O’Keeffe is also supported by the HRB Emerging Investigator Award (EIA-FA-2019-007 SCaRLeT) and a UK Medical Research Council Population Health Scientist fellowship (MR/M014509/1). Dr Bell is supported by the Elizabeth Blackwell Institute for Health Research, University of Bristol and the Wellcome Trust Institutional Strategic Support Fund (204813/Z/16/Z). Drs Davey Smith and Tilling work in a unit funded by the UK MRC (MC_UU_00011/1 and MC UU 00011/3) and the University of Bristol. These funding sources had no role in the design and conduct of this study. This publication is the work of the authors, and Dr O’Neill will serve as guarantor for the contents of this article.

### Disclosures

None.

## Supplementary Material


